# Vascular Dementia: From Pathophysiology to Therapeutic Frontiers

**DOI:** 10.3390/jcm14186611

**Published:** 2025-09-19

**Authors:** Han-Mo Yang

**Affiliations:** Division of Cardiology, Department of Internal Medicine, Seoul National University Hospital, Seoul 03080, Republic of Korea; hanname@gmail.com; Tel.: +82-2-2072-4184

**Keywords:** vascular dementia, vascular cognitive impairment, cerebral small vessel disease, neuroinflammation, endothelial dysfunction, blood–brain barrier, glymphatic system, biomarkers

## Abstract

Vascular dementia (VaD) represents the second-most common dementia type after Alzheimer’s disease since it results from complications of cerebrovascular disease. Mixed pathologies combining vascular and neurodegenerative processes are the rule rather than exception in elderly dementia patients. The condition known as VaD includes various types of vascular damage that affect both large and small blood vessels in the brain which results in cerebral hypoperfusion, blood–brain barrier disruption, glymphatic dysfunction, and molecular cascades causing neuronal damage. The mechanisms of VaD include endothelial dysfunction, oxidative stress, chronic neuroinflammation, impaired glymphatic clearance, white matter demyelination, and synaptic failure. The disease susceptibility of individuals depends on genetic factors which include NOTCH3 mutations and vascular risk polymorphisms. The diagnostic field uses neuroimaging tools and fluid biomarkers such as neurofilament light chain, inflammatory markers, and Aβ/tau ratios for mixed pathology. The current practice of vascular risk management combines with new therapeutic approaches that use phosphodiesterase inhibitors for cerebral perfusion and NLRP3 inflammasome inhibitors for neuroinflammation, senolytics for cellular senescence, and remyelination agents for white matter repair. However, the majority of new treatment methods remain investigational with limited Phase III data. Future medical treatment development will depend on precision medicine approaches which use biomarker-guided treatment selection and combination strategies targeting multiple pathological mechanisms.

## 1. Introduction

Dementia is a global public health priority, affecting over 55 million people worldwide, a number projected to nearly triple by 2050 [1,2]. While Alzheimer’s disease (AD) is the most prevalent form, vascular cognitive impairment (VCI) and its most severe form, vascular dementia (VaD), represent the second leading cause, accounting for approximately 15–20% of all dementia cases [3,4]. VaD is not a single entity but a clinically and neuropathologically heterogeneous syndrome where cognitive decline is causally related to cerebrovascular disease [5]. The underlying vascular pathologies can range from a single, strategically located infarct to multiple cortical/subcortical strokes, and, most commonly, diffuse white matter lesions and lacunes caused by cerebral small vessel disease (SVD) [6,7].

The traditional view of dementia is often segregated into neurodegenerative (like AD) and vascular causes. However, a paradigm shift has occurred, with growing recognition that mixed pathologies, particularly the coexistence of AD and cerebrovascular disease, are the rule rather than the exception in elderly dementia patients [8,9]. Cerebrovascular lesions not only contribute independently to cognitive decline but also lower the threshold for dementia in the presence of AD pathology, while conversely, amyloid deposition can compromise vascular integrity through cerebral amyloid angiopathy, creating a bidirectional pathological relationship [10,11]. This crosstalk highlights the critical importance of understanding the vascular contributions to cognitive impairment to develop effective prevention and treatment strategies for all-cause dementia.

The molecular underpinnings of VaD are intricate and involve a destructive cascade initiated by chronic cerebral hypoperfusion and ischemia. This leads to a breakdown of the blood–brain barrier (BBB), endothelial dysfunction, rampant oxidative stress, impaired glymphatic clearance, and sustained neuroinflammation [12,13]. These events converge to inflict damage on the most vulnerable cerebral tissues, particularly the subcortical white matter, leading to oligodendrocyte death, demyelination, axonal damage, and ultimately the disruption of critical neural networks essential for cognitive function [14,15].

Despite its prevalence and devastating impact, there are currently no approved disease-modifying therapies specifically for VaD. Current management is largely limited to the aggressive control of vascular risk factors such as hypertension, diabetes mellitus, hyperlipidemia, and smoking [16,17]. While crucial for prevention, these strategies have limited efficacy in reversing or halting the progression of established cognitive decline. Therefore, a deeper understanding of the specific molecular and cellular mechanisms driving VaD pathogenesis is paramount to identifying novel therapeutic targets.

This review aims to provide a comprehensive synthesis of the molecular mechanisms and pathophysiology underlying VaD. The review will first outline the major pathophysiological subtypes of VaD (Table 1). We will then dissect the core molecular pathways—from endothelial cell dysfunction and oxidative stress to neuroinflammation, glymphatic dysfunction, and white matter injury—that form the mechanistic basis of the disease. The role of genetic predispositions will be discussed. Subsequently, this paper will review the progress in developing sensitive and specific biomarkers, including advanced neuroimaging and fluid-based markers (Table 2), for improved diagnosis and patient stratification. Finally, the review will summarize current management approaches and explore the promising landscape of future therapeutic strategies aimed at directly targeting the molecular drivers of neuronal damage in VaD, while acknowledging the preliminary nature of many novel approaches and the need for rigorous clinical validation.

## 2. The Spectrum of Cerebrovascular Pathophysiology in VaD

The term VaD encompasses a range of cognitive syndromes caused by different types of cerebrovascular pathologies. The nature of the cognitive deficits often depends on the location, extent, and type of the underlying vascular brain injury. The main categories are summarized in Table 1 and discussed below.

### 2.1. Post-Stroke Dementia (PSD)

PSD is defined as any dementia that develops within a year following a clinical stroke [18]. It can result from a single, large infarct in a strategically important brain region for cognition, such as the thalamus, hippocampus, or angular gyrus [19,20]. More commonly, it arises from the cumulative effect of multiple cortical or subcortical infarcts (multi-infarct dementia) which progressively degrade neural circuits [21]. The risk of developing PSD is influenced by stroke severity, location, stroke recurrence, and the pre-stroke cognitive status of the individual [22]. The molecular sequelae of ischemic stroke, including excitotoxicity, peri-infarct inflammation, and glial scar formation, are central to the neuronal loss and circuit disruption seen in PSD [23].

**Table 1:** Major Clinicopathological Subtypes of Vascular Dementia.

Subtype	Primary Underlying Pathology	Key Neuroimaging Features	Typical Clinical Presentation	References
**Post-Stroke Dementia (PSD)**	Single strategic infarct or multiple large-vessel infarcts (Multi-Infarct Dementia).	Cortical/subcortical infarcts in major arterial territories; focal atrophy.	Stepwise or abrupt cognitive decline following a clinical stroke; deficits depend on infarct location.	[18,19,20,21,22]
**Subcortical Ischemic VaD (SIVD)**	Cerebral small vessel disease (SVD): arteriolosclerosis, lipohyalinosis.	Confluent white matter hyperintensities (WMHs), multiple lacunar infarcts, cerebral microbleeds, enlarged perivascular spaces.	Insidious onset and gradual progression; prominent executive dysfunction, psychomotor slowing, apathy, gait disturbance.	[24,25,26,27,28,29,30]
**CADASIL**	Autosomal dominant mutations in the *NOTCH3* gene causing VSMC degeneration.	Confluent WMHs with characteristic anterior temporal lobe involvement; multiple lacunes.	Migraine with aura (early), recurrent strokes, mood disturbances, progressive cognitive decline in young to mid-adulthood.	[31,32,33,34,35]
**Mixed Dementia (VaD + AD)**	Coexistence of cerebrovascular disease (any type) and Alzheimer’s pathology (plaques & tangles).	Features of both VaD (e.g., infarcts, WMHs) and AD (e.g., medial temporal atrophy).	Often amnestic presentation (like AD) but with additional features of vascular disease; typically more rapid decline.	[8,9,10,11]

VaD, Vascular dementia; AD, Alzheimer’s disease; CADASIL, Cerebral autosomal dominant arteriopathy with subcortical infarcts and leukoencephalopathy; NOTCH3, Neurogenic locus notch homolog protein 3; VSMC, Vascular smooth muscle cell.

### 2.2. Subcortical Ischemic Vascular Dementia (SIVD)

SIVD, often referred to as Binswanger’s disease in its severe form, is arguably the most common form of VaD [24]. It is primarily a manifestation of SVD, a pathology of the brain’s small perforating arteries and arterioles [7,25]. Chronic hypertension and other vascular risk factors lead to lipohyalinosis and arteriolosclerosis of these small vessels, resulting in reduced vessel compliance and cerebral blood flow (CBF) [26]. The hallmark neuropathological features are diffuse, confluent white matter hyperintensities (WMHs) on T2-weighted Magnetic-resonance Imaging (MRI), lacunar infarcts in deep brain structures, cerebral microbleeds, and enlarged perivascular spaces [27,28]. SIVD typically presents with a “subcortical syndrome” characterized by prominent executive dysfunction, slowed processing speed, and gait disturbances, with memory being relatively preserved in the early stages [29]. The underlying pathology is one of chronic hypoperfusion and ischemia of the deep white and grey matter, which are watershed areas highly vulnerable to drops in perfusion pressure [30].

### 2.3. Hereditary Forms and CADASIL

While most VaD is sporadic, several monogenic forms exist, offering unique insights into the direct consequences of specific vascular gene mutations. The most common hereditary form is Cerebral Autosomal Dominant Arteriopathy with Subcortical Infarcts and Leukoencephalopathy (CADASIL) [31]. CADASIL is caused by stereotyped mutations in the *NOTCH3* gene on chromosome 19, which encodes a transmembrane receptor crucial for vascular smooth muscle cell (VSMC) differentiation and survival [32,33]. The mutations lead to the abnormal accumulation of Notch3 extracellular domain protein in the vessel walls of small arteries, leading to VSMC degeneration, vessel fibrosis, and severe stenosis [34]. This results in recurrent subcortical strokes and a progressive leukoencephalopathy, typically leading to dementia and disability in mid-adulthood [35]. Other rare hereditary forms include CARASIL caused by *HTRA1* mutations [36].

## 3. Core Molecular Mechanisms of Neuronal Injury

**Figure 1:**
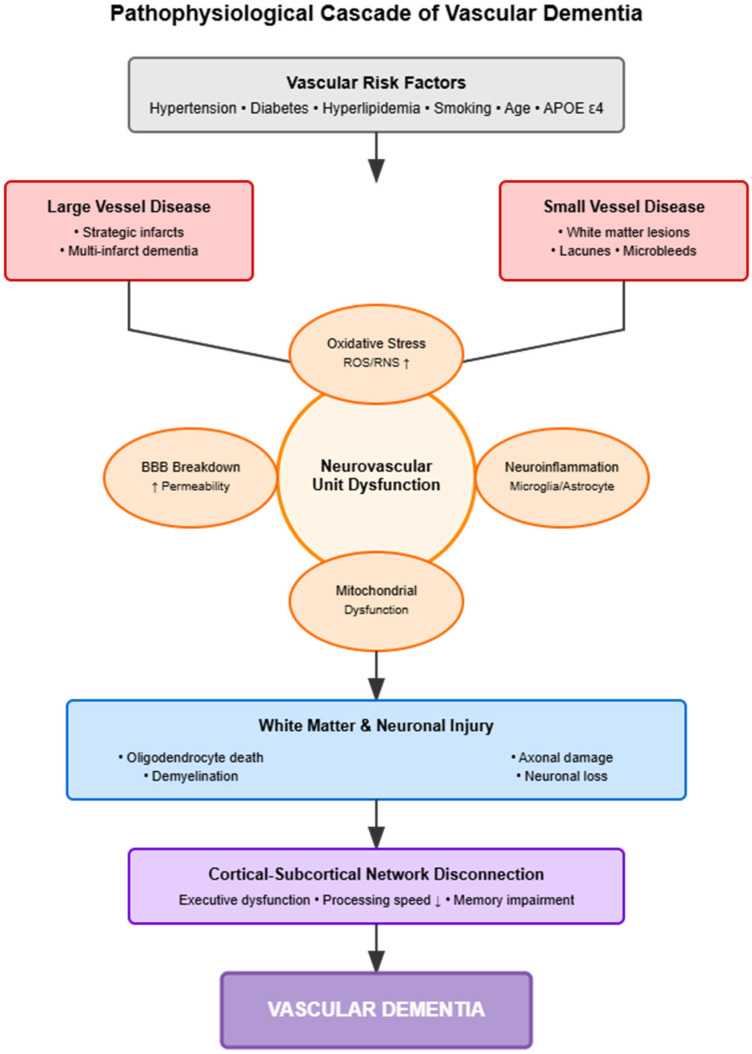
Pathophysiological Cascade of Vascular Dementia. The cascade from vascular risk factors through molecular mechanisms to clinical dementia. Risk factors initiate large/small vessel pathology, causing neurovascular dysfunction (BBB breakdown, oxidative stress, inflammation, and mitochondrial dysfunction), leading to tissue injury and network disconnection manifesting as cognitive impairment.

Regardless of the primary vascular insult, a common set of downstream molecular and cellular pathways are activated, creating a hostile microenvironment that drives the progressive neurodegeneration seen in VaD (Figure 1).

### 3.1. Endothelial Dysfunction and BBB Breakdown

The neurovascular unit (NVU) is a sophisticated multicellular complex comprising endothelial cells, pericytes, astrocytes, and neurons that collectively maintain brain homeostasis [37]. In VaD, the endothelium is a primary target. Chronic hypoperfusion and vascular risk factors trigger endothelial activation and dysfunction. This is characterized by a switch from a quiescent, anti-thrombotic state to a pro-inflammatory, pro-thrombotic phenotype [38]. Endothelial cells upregulate the expression of adhesion molecules like vascular cell adhesion molecule-1 (VCAM-1) and intercellular adhesion molecule-1 (ICAM-1), facilitating leukocyte infiltration into the brain parenchyma [39]. Furthermore, the production of the vasodilator nitric oxide (NO) by endothelial NO synthase (eNOS) is impaired, while the production of vasoconstrictor endothelin-1 is increased, further compromising CBF [40,41].

A critical consequence of endothelial dysfunction is the breakdown of the BBB. Tight junctions and adherent junctions between endothelial cells are disassembled through the proteolytic activity of matrix metalloproteinases (MMPs), particularly MMP-9, released from activated endothelial cells and astrocytes [42,43]. This leads to increased BBB permeability, allowing serum proteins like albumin, fibrinogen, and thrombin to leak into the brain interstitium [12]. These proteins are neurotoxic: albumin can trigger astrocytic activation and inflammation, while fibrinogen can deposit and impair remyelination [44,45]. This “leaky” BBB is a consistent and early finding in patients with SVD and VaD, and the degree of leakage correlates with the severity of white matter injury [46].

### 3.2. Oxidative Stress

The brain is highly susceptible to oxidative stress due to its high metabolic rate, high lipid content, and relatively low antioxidant capacity [47]. In VaD, chronic hypoperfusion-reperfusion cycles in the microvasculature create a state of metabolic stress, leading to the overproduction of reactive oxygen species (ROS) and reactive nitrogen species (RNS) [48]. Key sources of ROS include NADPH oxidase (NOX) enzymes, particularly NOX2 and NOX4 expressed in endothelial cells, microglia, and neurons, as well as uncoupled eNOS and dysfunctional mitochondria [49,50].

ROS inflict widespread damage by oxidizing lipids (lipid peroxidation), proteins, and nucleic acids [51]. Lipid peroxidation of cell membranes compromises their integrity and function. Oxidative damage to DNA can trigger cell cycle arrest or apoptosis. This rampant oxidative stress overwhelms the brain’s antioxidant defense systems (e.g., superoxide dismutase, catalase, glutathione peroxidase), creating a vicious cycle of further cellular damage and inflammation [52]. Markers of oxidative damage are significantly elevated in the brain and cerebrospinal fluid (CSF) of VaD patients [53].

### 3.3. Neuroinflammation and Immune Activation

Once considered a sterile process, it is now clear that a chronic, non-resolving inflammatory response is a key driver of VaD pathology [13,54]. BBB breakdown and endothelial activation permit the infiltration of peripheral immune cells. Concurrently, resident immune cells of the central nervous system (CNS), primarily microglia and astrocytes, become chronically activated [55].

Initially, microglial activation is a protective response aimed at clearing debris. However, under chronic ischemic stress, microglia adopt a persistent pro-inflammatory phenotype, releasing a barrage of cytotoxic mediators, including pro-inflammatory cytokines (TNF-α, IL-1β, IL-6), chemokines, ROS, and glutamate [56,57]. Astrocytes also become reactive, a process known as astrogliosis, contributing to the inflammatory milieu and forming a glial scar that can inhibit axonal regeneration and remyelination [58]. This sustained inflammatory state, fueled by signals from the damaged endothelium and leaky BBB, directly contributes to oligodendrocyte apoptosis, demyelination, and neuronal death [59,60]. The inflammasome, particularly the NLR family pyrin domain containing 3 (NLRP3) inflammasome, has been identified as a key platform for processing and releasing IL-1β in response to vascular stress signals, making it a potential therapeutic target [61].

### 3.4. White Matter Injury: Oligodendrocyte Death and Demyelination

The subcortical white matter is the “battleground” in SIVD. Oligodendrocytes, the myelin-producing cells of the CNS, are exceptionally vulnerable to ischemic and inflammatory insults [14,62]. They have a very high metabolic rate and limited antioxidant defenses, making them susceptible to damage from hypoperfusion and oxidative stress [63].

The hostile microenvironment created by inflammation, ROS, and excitotoxicity triggers oligodendrocyte apoptosis [64]. Furthermore, oligodendrocyte precursor cells (OPCs), which are present in the adult brain and capable of differentiating to replace lost oligodendrocytes, are also impaired. The inflammatory environment can arrest OPC maturation, preventing effective remyelination [65,66]. The result is a net loss of myelin (demyelination) and damage to the underlying axons. This disconnects cortical and subcortical regions, disrupting the large-scale neural networks required for higher-order cognitive functions like executive control and processing speed, leading directly to the clinical syndrome of SIVD [15,67].

### 3.5. Glymphatic Dysfunction and Impaired Waste Clearance

The glymphatic system functions as a newly identified paravascular pathway which extends throughout the brain to remove amyloid-β and tau proteins and other metabolic waste products from brain tissue [68]. This system relies on the convective flow of CSF through periarterial spaces, exchange with interstitial fluid facilitated by aquaporin-4 (AQP4) water channels on astrocytic endfeet, and drainage along perivenous spaces [69]. The combination of various factors in VaD disrupts glymphatic function which leads to dangerous waste accumulation that intensifies neurodegenerative damage.

The glymphatic clearance process becomes impaired because of cerebrovascular pathology through multiple pathways. The stiffened arteries and decreased pulsatile flow in hypertensive small vessel disease reduce the pressure that drives CSF to enter the periarterial space [70]. The loss of vascular smooth muscle cells and pericytes in SVD causes vasomotion to decrease which worsens the perivascular pumping mechanism [71]. The redistribution of AQP4 from astrocytic endfeet to the parenchymal membrane (loss of AQP4 polarization) and reactive astrogliosis disrupts the essential water transport needed for glymphatic exchange [72].

The blood–brain barrier disruption in VaD prevents the glymphatic system from functioning properly. The process of protein leakage into tissues and the inflammatory response around blood vessels creates blockages in perivascular spaces while blood-derived fibrinogen proteins physically obstruct drainage routes [73]. The glymphatic system operates under circadian control and reaches its peak activity during sleep periods especially during non-rapid eye movement (REM) sleep stages [74]. Sleep disturbances which frequently affect VaD patients create a self-reinforcing cycle that worsens glymphatic dysfunction while allowing pathological substances to build up [75].

Glymphatic failure in VaD produces effects which go beyond the accumulation of waste products. The high rate of mixed pathology in VaD patients can be explained by impaired amyloid-β clearance because vascular dysfunction speeds up amyloid deposition [76]. The body fails to eliminate inflammatory mediators and damage-associated molecular patterns (DAMPs) which leads to their accumulation and causes ongoing neuroinflammation. The presence of neurotoxic metabolites in the brain leads to direct damage of neurons and oligodendrocytes which affects watershed areas that experience reduced blood flow [77].

### 3.6. Temporal Evolution of Molecular Cascades

The correct order of VaD disease progression needs to be understood because it helps researchers find optimal treatment times and create treatments that match disease stages. The molecular sequence of events follows a standard pattern, but the time course depends on the degree of vascular injury and individual resistance factors [78]. The first detectable signs of the disease appear before patients show symptoms and these changes start with endothelial dysfunction and small increases in BBB permeability [79]. Endothelial cells in this preclinical stage produce less nitric oxide while showing higher adhesion molecule expression and initial tight junction breakdown. The first signs of glymphatic dysfunction appear because of decreased arterial pulsatility and the loss of pericytes during the early stages of the disease [80]. The tests conducted at this stage demonstrate both enlarged perivascular spaces and minimal white matter changes on diffusion tensor imaging (DTI) and fluid biomarkers indicate small increases in endothelial activation markers [81].

The disease progresses to the early symptomatic phase (mild cognitive impairment stage) because BBB breakdown becomes more severe and albumin begins to leak into brain parenchyma [82]. The body experiences elevated oxidative stress when antioxidant defenses reach their maximum operational capacity. The initial protective response of microglial activation develops into a long-lasting pro-inflammatory state which produces continuous cytokine and reactive oxygen species release [83]. The first sign of oligodendrocyte stress becomes apparent through early apoptosis that specifically attacks the vulnerable periventricular areas of the brain. The clinical presentation of patients includes executive dysfunction and processing speed deficits while neuroimaging shows white matter hyperintensities and early lacunes [84].

In established VaD, the pathological cascades reach their full expression. Chronic neuroinflammation becomes self-sustaining through activation of the NLRP3 inflammasome and other inflammatory platforms [85]. The brain shows its most severe oligodendrocyte death and demyelination in the frontal and periventricular white matter areas. The extent of axonal damage becomes severe enough to produce detectable increases in neurofilament light chain levels which appear in both CSF and plasma samples [86]. The severe condition of glymphatic failure leads to the accumulation of various toxic substances. The damaged white matter tracts cause secondary neurodegeneration to spread throughout cortical areas which results in network-wide dysfunction [87].

The advanced stage of the disease shows permanent structural damage through tissue loss and gliosis and complete breakdown of essential neural networks [88]. The brain demonstrates accelerated aging signs through cellular senescence which occurs when compensatory mechanisms reach their limits. The molecular chain reactions create an unfavorable setting which prevents all attempts to fix or regenerate tissues thus making treatments for late-stage diseases useless [89].

The established time frame provides essential information which enables researchers to study biomarkers and develop fresh therapeutic solutions. Early interventions that protect endothelial function and BBB integrity can stop the progression of disease but treatment at later stages needs to target multiple disease mechanisms at once [90].

## 4. Genetic Risk Factors

Beyond the well-established monogenic form of CADASIL (*NOTCH3*), research has sought to identify genetic risk factors for the more common, sporadic forms of VaD. The genetic architecture appears complex, involving multiple genes of small effect, many of which are related to vascular health, inflammation, and lipid metabolism.

The apolipoprotein E (APOE) ε4 allele, the strongest genetic risk factor for AD, has also been implicated in VaD, although its role is more controversial [91]. Some studies suggest APOE ε4 increases the risk of PSD and may lower the threshold for dementia in the presence of vascular pathology, possibly by exacerbating BBB breakdown and promoting cerebral amyloid angiopathy (CAA) [92,93]. Emerging preclinical and early human evidence indicates that APOE ε4 may also impair glymphatic function, providing another potential linking this allele to both vascular and Alzheimer pathology [94].

Polymorphisms in the gene encoding methylenetetrahydrofolate reductase (MTHFR), which can lead to hyperhomocysteinemia—a known vascular risk factor—have been associated with SVD and VaD in some populations [95]. Homocysteine can promote endothelial dysfunction and oxidative stress. Genes involved in inflammatory pathways, such as *IL-6* and *TNF-α*, have also been investigated, with some polymorphic variants showing a modest association with VaD risk [96].

More recently, genome-wide association studies (GWAS) have begun to identify novel loci associated with SVD imaging markers, such as WMHs. Loci near genes involved in endothelial function, blood pressure regulation, and extracellular matrix integrity have been pinpointed, though their direct link to a clinical VaD diagnosis requires further validation [97,98]. The genetic landscape of sporadic VaD is an active area of research, with larger, more diverse cohorts needed to uncover robust associations.

## 5. Diagnosis and the Quest for Biomarkers

The diagnosis of VaD is challenging due to its clinical heterogeneity and frequent overlap with AD. Diagnosis relies on a combination of clinical assessment (evidence of cognitive decline and cerebrovascular disease) and, crucially, neuroimaging. The development of reliable biomarkers is a major goal to improve diagnostic accuracy, track disease progression, and serve as surrogate endpoints in clinical trials.

### 5.1. Neuroimaging Biomarkers

**MRI** is the cornerstone of VaD diagnosis. Standard MRI sequences can visualize the macroscopic signatures of cerebrovascular disease [25,99].

- **T2-weighted and FLAIR sequences** are highly sensitive for detecting WMHs, which are a key feature of SVD. The volume and progression of WMHs are correlated with cognitive decline [27].
- **T1-weighted sequences** identify chronic lacunar and cortical infarcts and can be used to measure brain atrophy, which is also an indicator of disease severity.
- **Susceptibility-weighted imaging (SWI) or T2-gradient echo sequences** are exquisitely sensitive for detecting cerebral microbleeds, which are markers of fragile, leaky small vessels [28].

Advanced MRI techniques provide deeper, quantitative insights into the microstructural and physiological consequences of vascular disease.

- **Diffusion Tensor Imaging (DTI)** measures the random motion of water molecules to assess white matter microstructural integrity. Metrics like fractional anisotropy (FA) and mean diffusivity (MD) can detect subtle axonal damage and demyelination within WMHs and even in normal-appearing white matter, often before changes are visible on conventional MRI [100,101].
- **Arterial Spin Labeling (ASL)** and **Dynamic Susceptibility Contrast (DSC)-MRI** are perfusion imaging techniques that can non-invasively quantify CBF, providing a direct measure of cerebral hypoperfusion [102].
- **Dynamic Contrast-Enhanced (DCE)-MRI** can be used to quantify BBB permeability, providing a direct in vivo marker of BBB breakdown [46].

### 5.2. Fluid Biomarkers

Fluid biomarkers, measured in CSF or blood, offer a less invasive and potentially more scalable approach for diagnosis and monitoring. The search for VaD-specific fluid biomarkers is ongoing, with a focus on markers reflecting the core pathologies (Table 2).

- **Markers of Neuronal and Axonal Injury:** CSF and plasma levels of neurofilament light chain (NfL), a marker of axonal damage, are elevated in VaD, often to a greater extent than in AD [103].
- **Markers of Inflammation:** Pro-inflammatory cytokines, markers of glial activation (e.g., GFAP, YKL-40, sTREM2), and endothelial adhesion molecules (e.g., VCAM-1) are being investigated in both CSF and blood, with many showing elevated levels in VaD patients [54,104].
- **Markers of BBB and Endothelial Dysfunction:** The CSF/serum albumin ratio (Qalb) is a classic marker of BBB integrity and is often elevated in VaD [12]. Soluble forms of tight junction proteins and markers of pericyte injury are also emerging as candidate biomarkers [105].

The main challenge is specificity, as many of these markers are also altered in other neurodegenerative and inflammatory conditions. A panel of multiple biomarkers, combining plasma markers like p-tau for AD pathology and NfL for axonal injury, will likely be necessary for accurate differential diagnosis [103].

### 5.3. Biomarker Dynamics Across Disease Stages

The progression of biomarkers in VaD follows a specific timeline which mirrors the natural progression of disease pathology. The appropriate selection of biomarkers depends on disease stage and disease progression monitoring because researchers need to understand these biological processes [106] (Table 3).

The first detectable preclinical stage changes become detectable through DCE-MRI and CSF/serum albumin ratio measurements that show minimal BBB permeability changes [107]. The plasma levels of endothelial activation markers VCAM-1 and ICAM-1 start to increase. The advanced DTI metrics demonstrate their ability to detect white matter microstructural changes which occur before WMHs become visible to standard imaging techniques. The glymphatic system shows initial signs of breakdown according to DTI-ALPS (Diffusion Tensor Image Analysis Along the Perivascular Space) measurements [108].

Neuroimaging biomarkers become more important during the prodromal/MCI stage because WMH volume continues to increase while lacunes begin to develop [109]. The CSF shows elevated levels of inflammatory markers which include GFAP, YKL-40 and sTREM2. The levels of Neurofilament light chain start to rise because of continuous axonal damage. The brain areas that depend on watershed regions for blood supply show measurable blood flow reduction according to perfusion imaging [110].

In established dementia, all biomarker categories show marked abnormalities. The imaging results show white matter destruction throughout the brain together with multiple lacunes and significant brain tissue loss [111]. The levels of fluid biomarkers achieve equilibrium points at which NfL demonstrates the most direct relationship with clinical disease severity. Notably, the rate of biomarker change may be more informative than absolute levels for predicting progression [112].

### 5.4. Clinical Utility of Biomarkers for Diagnosis and Prognosis

Biomarkers in VaD provide various clinical functions which include early disease detection and differential diagnosis and both prognostic assessment and treatment monitoring. The diagnostic worth of biomarkers depends on their particular type and the current stage of disease development (Table 2 and Table 3).

Neuroimaging functions as the main diagnostic instrument for medical purposes. The combination of WMH burden, lacunes, and microbleeds on MRI supports differentiation from healthy aging; diagnostic performance varies across cohorts, definitions, and imaging protocol, and combining markers improves diagnostic confidence [113]. The medical process of frontotemporal dementia (FTD) diagnosis requires additional testing to identify the condition apart from AD. The CSF Aβ42/40 ratio combined with p-tau measurements helps scientists identify mixed pathology which affects about 40% of VaD patients who receive clinical diagnoses [114]. The diagnostic accuracy of MRI improves through the use of advanced imaging techniques such as DTI and ASL perfusion which enable the detection of early-stage diseases when standard MRI results remain unclear [115].

The demand for prognostic applications continues to rise because they help doctors deliver improved patient guidance and support clinical trial development. The risk of dementia conversion becomes 1.5 times higher when baseline WMH volume and progression rate increase by 10 mL [116]. Plasma NfL levels serve as an accessible prognostic tool because patients with levels above 40 pg/mL experience accelerated cognitive deterioration and elevated death risk [117]. The combination of imaging data with fluid biomarkers produces enhanced predictive results because new models achieve AUC values ranging from 0.85 to 0.90 for predicting 2-year cognitive changes [118].

For treatment monitoring, biomarkers offer objective measures of therapeutic response. The permeability changes in the BBB that DCE-MRI measures become detectable for treatment effects within 3–6 months [119]. GFAP and other inflammatory markers demonstrate their ability to react to anti-inflammatory treatments. Serial NfL measurements have the potential to function as substitute endpoints in clinical trials which could speed up drug development processes [120].

### 5.5. Biomarkers as Windows into Pathological Mechanisms

Beyond their clinical utility, biomarkers provide insights into dominant pathological processes in individual patients, enabling mechanistic stratification for precision medicine approaches [121].

The markers VCAM-1, ICAM-1 and von Willebrand factor help to identify patients with primary vascular disease who need vascular-targeted treatments the most [122]. The inflammatory biomarkers are also helpful in identifying patients who have neuroinflammation as their main condition because these patients might benefit from anti-inflammatory treatments. Elevated NLRP3 inflammasome components suggest suitability for inflammasome inhibitors [123].

The combination of DTI-ALPS and CSF flow imaging enables doctors to detect patients who need sleep optimization or AQP4 function-targeting therapies [124]. The presence of myelin basic protein and oligodendrocyte-specific proteins indicates that remyelination approaches may be beneficial for treating white matter injury [125].

The mechanistic profiling system allows doctors to choose specific treatments and combination therapies through analysis of individual disease patterns rather than depending on clinical symptoms [126].

## 6. Current and Future Therapeutic Strategies

**Figure 2:**
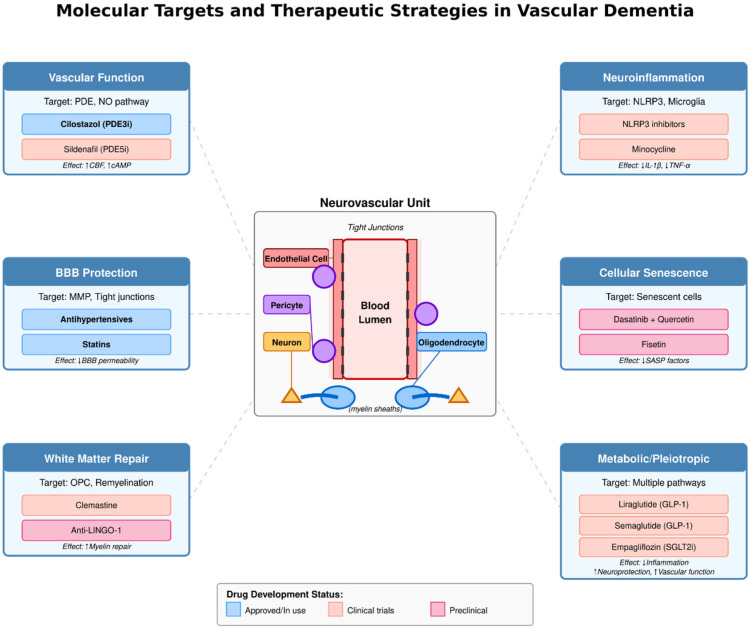
**Molecular Targets and Therapeutic Strategies in Vascular Dementia** Therapeutic targets and approaches in vascular dementia include: (1) Vascular function—phosphodiesterase (PDE) inhibitors cilostazol (PDE3i) and sildenafil (PDE5i) enhance cerebral blood flow; (2) Neuroinflammation—NLRP3 (NOD-like receptor protein 3) inhibitors and minocycline reduce interleukin-1β (IL-1β) and tumor necrosis factor-α (TNF-α); (3) Blood–brain barrier (BBB) protection—antihypertensives and statins preserve tight junctions; (4) Cellular senescence—senolytics dasatinib + quercetin and fisetin decrease senescence-associated secretory phenotype (SASP) factors; (5) White matter repair—clemastine and anti-LINGO-1 promote oligodendrocyte precursor cell (OPC) differentiation and remyelination; (6) Metabolic/pleiotropic—glucagon-like peptide-1 (GLP-1) agonists liraglutide, semaglutide, and sodium–glucose cotransporter-2 inhibitor (SGLT2i) empagliflozin improve vascular and neuroprotection. Drug development stage: blue, approved; peach, clinical trials; pink, preclinical.

**Figure 3:**
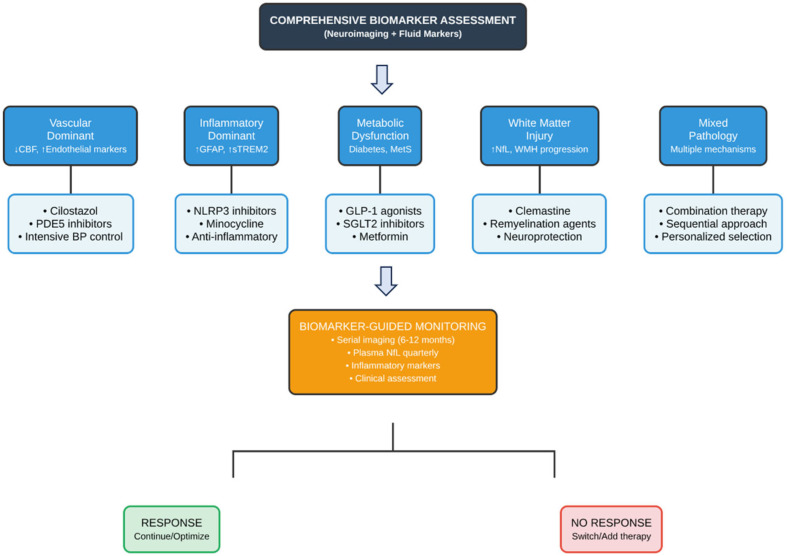
**Decision Tree for VaD Therapeutic Selection** A clinical decision algorithm for personalized treatment selection in vascular dementia based on biomarker-guided pathological profiling. The flowchart begins with comprehensive biomarker assessment combining neuroimaging (MRI, DTI, perfusion imaging) and fluid markers (CSF, plasma). Based on the dominant pathological mechanism identified, patients are stratified into five therapeutic pathways: (1) Vascular dominant pathology with reduced cerebral blood flow and elevated endothelial markers—treated with phosphodiesterase inhibitors and intensive blood pressure control; (2) Inflammatory dominant pathology with elevated glial markers—targeted with anti-inflammatory approaches; (3) Metabolic dysfunction including diabetes and metabolic syndrome—managed with metabolic modulators; (4) White matter injury with elevated neurofilament light chain and progressive white matter hyperintensities—addressed with remyelination agents; (5) Mixed pathology requiring combination or sequential therapeutic approaches. Following treatment initiation, biomarker-guided monitoring includes serial imaging every 6−12 months, quarterly plasma neurofilament measurements, and regular clinical assessments. Treatment response determines continuation and optimization versus switching or adding alternative therapies. Green indicates positive response pathway; red indicates non-response requiring treatment modification. BP, Blood pressure; CBF, Cerebral blood flow; CSF, Cerebrospinal fluid; DTI, Diffusion tensor imaging; GFAP, Glial fibrillary acidic protein; GLP-1, Glucagon-like peptide-1; MetS, Metabolic syndrome; MRI, Magnetic resonance imaging; NfL, Neurofilament light chain; NLRP3, NLR family pyrin domain containing 3; PDE5, Phosphodiesterase type 5; SGLT2, Sodium-glucose cotransporter 2; sTREM2, Soluble triggering receptor expressed on myeloid cells 2; VaD, Vascular dementia; WMH, White matter hyperintensities.

It is important to acknowledge at the outset that despite decades of research, no disease-modifying therapies are specifically approved for VaD in the United States, and the therapeutic landscape remains challenging. The following sections present both current management strategies and new investigational therapeutic methods which have not received sufficient Phase III clinical trial evidence [127] (Figure 2 and Figure 3).

### 6.1. Aggressive Management of Vascular Risk Factors

The cornerstone of VaD management is prevention and the strict control of modifiable vascular risk factors [16]. The following are key examples of risk factor management strategies, not an exhaustive list of available interventions [128].

**Table 2:** Selected Current and Emerging Biomarkers for Vascular Dementia.

Biomarker Type	Marker	What It Measures	Utility in VaD	Disease Stage	Mechanistic Pathway	References
**Neuroimaging**	**White Matter Hyperintensities (WMH) on FLAIR MRI**	Chronic ischemic white matter damage, gliosis, demyelination	Core diagnostic feature of SVD; volume & progression correlate with cognitive decline	Early to Advanced	Hypoperfusion, BBB breakdown	[27,99,113]
	**Diffusion Tensor Imaging (DTI)/Free-Water Imaging**	White matter microstructural integrity (axonal damage, neuroinflammation)	Detects early, subtle white matter damage; sensitive to change	Preclinical to Early	Axonal injury, inflammation	[100,101,115]
	**Perfusion MRI (e.g., ASL)**	Cerebral blood flow (CBF)	Quantifies chronic hypoperfusion, a key upstream driver	All stages	Vascular dysfunction	[102,110]
	**Dynamic Contrast-Enhanced (DCE)-MRI**	Blood–brain barrier (BBB) permeability	Direct in vivo measure of BBB leakiness, an early event	Preclinical to Early	Endothelial dysfunction	[46,107,119]
	**DTI-ALPS (Diffusion Tensor Image Analysis Along the Perivascular Space)**	Glymphatic function	Assesses perivascular drainage and waste clearance	Preclinical to Established	Glymphatic dysfunction	[108,124]
**CSF Fluid**	**Neurofilament Light Chain (NfL)**	Axonal injury and degeneration	Elevated in VaD; reflects ongoing neuronal damage	Early to Advanced	Axonal degeneration	[86,103,117]
	**CSF/Serum Albumin Ratio (Qalb)**	BBB integrity	Established marker of BBB breakdown	All stages	BBB dysfunction	[12,82,107]
	**GFAP/YKL-40/sTREM2**	Astrocyte and microglia activation	Markers of neuroinflammation; elevated in VaD	Early to Established	Neuroinflammation	[54,83,104]
	**Aβ42/40 ratio**	Concurrent AD pathology	Identifies mixed dementia cases	All stages	Amyloid pathology	[114,127]
	**p-tau (181, 217)**	Tau pathology	Distinguishes AD from pure VaD	All stages	Neurodegeneration	[103,114]
**Blood Fluid**	**Plasma NfL**	Systemic measure of axonal injury	Highly correlated with CSF NfL; minimally invasive	Early to Advanced	Axonal degeneration	[103,117,120]
	**Plasma GFAP**	Astrocyte reactivity	Elevated in VaD and SVD	Early to Established	Astrogliosis, BBB damage	[104,123]
	**Plasma p-tau species**	Alzheimer’s disease co-pathology	Crucial for differential diagnosis	All stages	Tau pathology	[103,114,127]
	**Plasma Aβ42/40 ratio**	Amyloid pathology	Emerging marker for mixed pathology	All stages	Amyloid accumulation	[127,128]

AD, Alzheimer’s disease; ALPS, Analysis Along the Perivascular Space; ASL, Arterial spin labeling; Aβ, Amyloid-beta; BBB, Blood–brain barrier; CBF, Cerebral blood flow; CSF, Cerebrospinal fluid; DCE-MRI, Dynamic contrast-enhanced magnetic resonance imaging; DTI, Diffusion tensor imaging; DTI-ALPS, Diffusion tensor image analysis along the perivascular space; FLAIR, Fluid-attenuated inversion recovery; GFAP, Glial fibrillary acidic protein; MRI, Magnetic resonance imaging; NfL, Neurofilament light chain; p-tau, Phosphorylated tau; Qalb, CSF/serum albumin ratio; sTREM2, Soluble triggering receptor expressed on myeloid cells 2; SVD, Small vessel disease; VaD, Vascular dementia; WMH, White matter hyperintensities; YKL-40, Chitinase-3-like protein 1.

**Table 3:** Stage-Specific Biomarker Profiles in Vascular Dementia.

Disease Stage	Clinical Features	Key Biomarker Changes	Therapeutic Implications	References
**Preclinical**	No symptoms; at-risk individuals	Subtle BBB permeability (DCE-MRI) Enlarged perivascular spaces Mild endothelial markers elevation Early glymphatic dysfunction	Prevention strategies; vascular risk factor control	[79,80,81,108]
**Prodromal/MCI**	Mild executive dysfunction	Progressive WMH Rising NfL and inflammatory markers Reduced CBF DTI abnormalities	Early intervention window; consider disease-modifying therapies	[84,109,110]
**Mild Dementia**	Clear functional impairment	Extensive WMH and lacunes Plateau inflammatory markers High NfL levels Brain atrophy begins	Combination therapies; symptom management	[86,87,111]
**Moderate-Severe**	Dependency in ADLs	Severe structural damage Network disconnection Maximal biomarker abnormalities	Supportive care; limited therapeutic options	[88,89,112]

ADLs, Activities of daily living; BBB, Blood–brain barrier; CBF, Cerebral blood flow; DCE-MRI, Dynamic contrast-enhanced magnetic resonance imaging; DTI, Diffusion tensor imaging; MCI, Mild cognitive impairment; NfL, Neurofilament light chain; WMH, White matter hyperintensities.

**Hypertension**: Antihypertensive treatment is paramount. Large clinical trials have shown that lowering blood pressure reduces the risk of stroke and may slow the progression of WMHs and cognitive decline [17,129]. The SPRINT-MIND trial suggested that intensive systolic blood pressure control (<120 mm Hg) could significantly reduce the risk of mild cognitive impairment [130].

**Diabetes Mellitus**: Tight glycemic control is important to prevent micro- and macrovascular complications that contribute to VaD. New antidiabetic agents like glucagon-like peptide-1 (GLP-1) receptor agonists may offer additional neuroprotective benefits [28,131].

**Hyperlipidemia**: Statin therapy, while primarily for stroke prevention, may have pleiotropic effects including improving endothelial function and reducing inflammation, though its direct benefit on cognition in established VaD requires more evidence [132].

**Lifestyle Modifications**: Smoking cessation, regular physical activity, a healthy diet (e.g., Mediterranean or DASH diet), and maintaining a healthy weight are all strongly recommended and have been shown to support brain and vascular health [133].

### 6.2. Symptomatic and Neuroprotective Approaches: An Expanding Frontier

The therapeutic approaches described below represent selected examples of promising strategies currently under investigation. It should be emphasized that most of these interventions lack definitive Phase III trial evidence and should be considered investigational [134].

Cholinesterase inhibitors (e.g., donepezil, galantamine) and the N-Methyl-D-aspartate.

(NMDA) receptor antagonist memantine, which are standard treatments for AD, have been tested in VaD with mixed and modest results [135]. Their limited efficacy underscores the different primary pathology and has propelled research into novel mechanisms that directly address the core tenets of VaD: endothelial dysfunction, neuroinflammation, white matter injury, and cellular senescence.

#### 6.2.1. Targeting Endothelial Function and Cerebral Perfusion

The compromised blood flow in VaD is a primary therapeutic target. Drugs that modulate vascular tone and health are under active investigation.

**Phosphodiesterase (PDE) Inhibitors**: Cilostazol, a PDE3 inhibitor, has demonstrated effects beyond its primary role in intermittent claudication. It possesses antiplatelet, anti-inflammatory, and vasodilatory properties that enhance cerebral blood flow. Although evidence is mixed, some trials, particularly in East Asia, have suggested that cilostazol can reduce the recurrence of stroke and may slow the progression of white matter lesions and cognitive decline in patients with SVD [136,137]. Its mechanism is thought to involve increasing cyclic AMP (cAMP) levels, which improves endothelial barrier function and reduces inflammation. Another class, PDE5 inhibitors (e.g., sildenafil), is also being explored preclinically for its ability to increase cerebral blood flow and have neuroprotective effects, although clinical data in VaD remains limited [138].

#### 6.2.2. Combating Neuroinflammation

Given the central role of chronic inflammation, targeting specific inflammatory pathways is a highly promising strategy.

**NLRP3 Inflammasome Inhibition**: The NLRP3 inflammasome is a key protein complex that, when activated by cellular stress signals (like those in VaD), triggers the release of potent pro-inflammatory cytokines IL-1β and IL-18. It is considered a master regulator of sterile inflammation. Preclinical models of SVD have shown that inhibiting NLRP3 can significantly reduce microglial activation, protect the BBB, and preserve white matter integrity [61]. Several brain-penetrant small molecule inhibitors of NLRP3 are now in early-phase clinical trials for various neurological conditions; their application to VaD remains investigational and will require dedicated trials [139].

**Targeting Microglia**: Minocycline, a tetracycline antibiotic with well-documented anti-inflammatory properties, can cross the BBB and suppress pro-inflammatory microglial activation. While it showed promise in preclinical models and small pilot studies [140], larger clinical trials have yielded mixed results, suggesting that more targeted approaches to modulate microglial phenotypes (e.g., shifting from a pro-inflammatory to a pro-resolving state) may be necessary.

#### 6.2.3. Cellular Senescence and Senolytics

A novel and exciting area of research is cellular senescence—a state of irreversible cell cycle arrest. Senescent endothelial cells and glial cells accumulate in the aging brain and contribute to VaD by secreting a cocktail of inflammatory proteins, known as the senescence-associated secretory phenotype (SASP) [141].

**Senolytics**: These are drugs that selectively induce apoptosis in senescent cells. In animal models of cognitive decline, clearing senescent cells with senolytics (such as the combination of Dasatinib and Quercetin, or Fisetin) has been shown to reduce neuroinflammation, improve neurogenesis, and restore cognitive function [142]. The rationale is that by removing the source of the chronic, pro-inflammatory SASP, the brain microenvironment can be restored to a healthier state. Clinical trials using senolytics for age-related conditions are underway, and their potential to treat VaD by “rejuvenating” the neurovascular unit is an area of intense investigation [143].

Research indicates that SGLT2 inhibitors which were first developed for diabetes treatment now show evidence of senolytic effects together with their established metabolic advantages. Preclinical studies demonstrate that SGLT2 inhibitors can decrease senescent cell numbers through their activation of AMPK and inhibition of mTOR signaling pathways [144]. The drugs show promise because they have proven safety records while multiple cardiovascular trials with cognitive assessments are currently underway [145].

#### 6.2.4. Promoting White Matter Repair and Remyelination

Since SIVD is fundamentally a disease of the white matter, strategies aimed at protecting and repairing oligodendrocytes are critical.

**Oligodendrocyte Protection and OPC Differentiation**: Research is focused on identifying compounds that both protect mature oligodendrocytes from ischemic and inflammatory death and stimulate OPCs to differentiate and create new myelin sheaths. Several molecular targets that inhibit OPC differentiation (e.g., LINGO-1, hyaluronan) have been identified, and blocking them can promote remyelination in disease models [66]. While most of this work has focused on multiple sclerosis, the principles are directly applicable to VaD. For instance, the antimuscarinic drug clemastine has been repurposed and shown to enhance remyelination, creating a potential therapeutic pathway for white matter repair [146].

#### 6.2.5. Metabolic and Pleiotropic Approaches

**GLP-1 Receptor Agonists**: Originally developed for type 2 diabetes, drugs like liraglutide and semaglutide have demonstrated potent neuroprotective effects in preclinical models of both AD and vascular injury. Their mechanisms are pleiotropic, including reducing inflammation, improving insulin signaling in the brain, decreasing oxidative stress, and supporting synaptic function [131,147]. Given the high comorbidity of metabolic syndrome and VaD, these agents are highly attractive candidates, and large-scale clinical trials assessing their cognitive outcomes are ongoing.

**Metformin**: Metformin serves as the initial antidiabetic medication which researchers now study for its brain-protecting properties beyond glucose control. The compound activates AMPK and simultaneously reduces neuroinflammation and shows potential to enhance autophagy and improve mitochondrial function [148]. Large observational studies suggest reduced dementia risk in diabetic patients on metformin, though prospective trials in VaD are needed [149].

**NAD+ precursors:** NAD+ precursors represent a promising therapeutic strategy for NAD+ metabolism. The combination of aging with vascular disease results in lower NAD+ levels which causes problems with cellular energy production and DNA repair mechanisms [150]. Preclinical research shows that NAD+ precursors NMN and NR protect blood vessels through their ability to boost endothelial function and reduce inflammation [151]. While research studies involving early human participants have demonstrated safety benefits together with cognitive advantages, more extensive large-scale clinical trials must be conducted for confirmation [152].

#### 6.2.6. Challenges and Realistic Expectations

While the therapeutic methods described above show promise, multiple obstacles exist to achieve clinical success from preclinical achievements. The various forms of VaD create a major challenge because people with different main disease types will react differently to treatment approaches [153]. The majority of clinical trials have employed wide participant selection criteria without biomarker-based subgrouping which could reduce treatment effectiveness for patients who would benefit from the intervention [154].

The blood–brain barrier acts as a barrier which prevents many potential compounds from entering the central nervous system. Researchers are currently testing drug delivery methods that combine focused ultrasound with nanoparticle formulations although these approaches exist only in experimental stages [155]. The advanced medical state and elderly condition of VaD patients requires close safety checks because they face higher risks of adverse effects and drug interactions [156].

The most important requirement is to start interventions before the current standard time. The point of clinical diagnosis indicates when major permanent tissue damage has already occurred. The research confirms that screening programs should identify high-risk individuals before they develop clinical symptoms, but more evaluation is required to determine their operational feasibility and cost-effectiveness [157].

#### 6.2.7. Targeting Glymphatic Function

Research into VaD has revealed new therapeutic approaches because of the recent discoveries about glymphatic system dysfunction. Sleep optimization serves as an immediate solution because the glymphatic system performs its best clearance work during sleep periods especially during slow-wave sleep [158]. CPAP therapy for sleep apnea patients with vascular disease improves glymphatic function while lowering their vascular risk according to research [159].

Scientists continue their research to develop pharmacological methods which will boost glymphatic system performance. Low-dose omega-3 fatty acid supplements show evidence that they can enhance AQP4 polarization and glymphatic flow [160]. Scientists are working on creating new substances which show promise to block AQP4 expression and function during preclinical research. The glymphatic pulsatility receives benefits from antihypertensive medications which improve arterial compliance according to research [161].

The future of VaD therapy will likely involve a precision medicine approach, using biomarkers to identify the dominant pathology in a given patient (e.g., inflammation vs. hypoperfusion) and tailoring treatment accordingly. Furthermore, combination therapies targeting multiple pathways simultaneously—for instance, an anti-inflammatory agent combined with a drug that promotes vascular health and remyelination—may hold the key to finally halting or reversing the progression of this devastating disease.

### 6.3. Framework for Therapeutic Implementation

The multiple treatment options for VaD demand a structured treatment plan because of its complex nature. The framework establishes its methodology through disease progression stages and primary disease mechanisms and treatment goals that can be achieved (Table 4).

### 6.4. Precision Medicine Approach to VaD Treatment

The future of VaD treatment will depend on precision medicine strategies which use specific treatment plans based on individual disease profiles instead of using only clinical symptoms for diagnosis. The treatment method needs complete biomarker evaluation to determine the main biological processes which will help choose the most suitable therapy [162].

The first step for treating patients with endothelial dysfunction and hypoperfusion (identified by reduced CBF on ASL-MRI and elevated endothelial markers) involves cilostazol or PDE5 inhibitors because these medications directly impact blood vessel function [163]. The treatment method that provides the most advantages to patients with elevated CSF sTREM2 and GFAP levels should implement anti-inflammatory approaches that utilize NLRP3 inhibitors or the repurposed medication minocycline [164].

The best results will emerge from implementing combination strategies which tackle various pathways. The treatment strategy for patients who have both vascular and inflammatory conditions in their blood vessels would involve PDE inhibitors to improve vascular function and GLP-1 agonists and anti-inflammatory drugs to enhance metabolic health [165]. The first step of sequential therapy requires vascular stabilization before doctors can proceed with neuroprotective treatments following acute injury management [166].

The monitoring system would enable doctors to modify treatment plans through biomarker data which shows how patients respond to their current therapy. The normalization of inflammatory markers would allow for lowering anti-inflammatory doses, but BBB leakage would require increased vascular protection measures [167]. The adaptive treatment strategy delivers the best possible results through the reduction in harmful side effects from treatments that do not provide benefits.

## 7. Conclusions and Future Directions

Vascular dementia is a complex and devastating disorder at the intersection of cerebrovascular health and neurodegeneration. Our understanding has evolved from a simplistic infarct-based model to a sophisticated appreciation of a molecular cascade initiated by chronic hypoperfusion and BBB failure, particularly in the context of SVD.

The core pathological pillars of endothelial dysfunction, oxidative stress, neuroinflammation, glymphatic dysfunction, and white matter injury work in concert to destroy vulnerable oligodendrocytes and neurons, leading to the disconnection of vital neural networks.

Research now shows that vascular and neurodegenerative diseases create a mutual relationship which helps explain dementia development in elderly people. The condition of vascular dysfunction leads to direct tissue damage and prevents the proper removal of neurotoxic proteins including amyloid-β. The presence of amyloid angiopathy together with tau pathology creates damage to blood vessels which weakens their structural integrity. The complex link between vascular and neurodegenerative elements results in a mixed pathology that needs treatment approaches which address both conditions simultaneously.

The path forward requires a multi-pronged approach. First, public health initiatives must continue to emphasize the aggressive control of vascular risk factors from midlife onwards, which remains our most effective tool for prevention. Second, the field must continue its quest for highly specific and sensitive biomarkers. A validated panel of imaging and fluid-based markers will be transformative, enabling early diagnosis, accurate prognosis, patient stratification for clinical trials, and objective monitoring of therapeutic response.

Third, and most critically, the development of disease-modifying therapies must be accelerated. However, we must maintain realistic expectations given the complexity of VaD and the disappointing translation of many preclinical successes. Future clinical trials should move beyond repurposed AD drugs and focus on agents that target the fundamental molecular pathways of VaD. Combination therapies that simultaneously target inflammation, oxidative stress, endothelial dysfunction, and glymphatic impairment may be necessary to combat the multifaceted nature of the disease. Furthermore, strategies aimed at promoting repair, such as enhancing BBB integrity or promoting remyelination, offer exciting new frontiers. Biomarkers serve as the most effective tool for medical progress when used to choose treatments through precision medicine strategies. The identification of specific disease mechanisms in individual patients enables healthcare providers to develop customized treatment plans which surpass conventional standardized medical protocols. The future of VaD treatment holds promising results because scientists keep working on new investigational treatments as they gain a better understanding of disease mechanisms and biomarkers and develop more advanced therapeutic approaches. By continuing to unravel the intricate molecular maze of VaD, we can move closer to the ultimate goal of preserving cognitive function in the face of vascular brain injury. The upcoming ten years will show whether experimental treatments under development will lead to actual medical advantages for the numerous patients suffering from this fatal disease.

## Figures and Tables

**Table 4:** Therapeutic Strategies Categorized by Mechanism and Stage.

Therapeutic Category	Specific Interventions	Mechanism	Classification	Optimal Disease Stage	Level of Evidence	References
**Vascular Risk Management**	Antihypertensives, Statins, Antiplatelet agents	Prevent further vascular injury	Disease-modifying (prevention)	All stages (critical in preclinical/early)	**Strong (Phase III data)**	[17,129,130,132]
**Metabolic Optimization**	GLP-1 agonists, SGLT2 inhibitors, Metformin	Multiple: anti-inflammatory, neuroprotective	Potentially both	Early to moderate	**Moderate (Phase II ongoing)**	[131,144,145,146,147,148,149]
**Cerebral Perfusion**	Cilostazol, PDE5 inhibitors	Enhance blood flow, reduce inflammation	Disease-modifying	Early to moderate	**Moderate (regional approval)**	[136,137,138,139]
**Anti-inflammatory**	NLRP3 inhibitors, Minocycline	Reduce neuroinflammation	Disease-modifying	Early to moderate	**Limited (Phase I/II)**	[61,85,137,140]
**Cellular Senescence**	Senolytics (D + Q, Fisetin)	Remove senescent cells	Disease-modifying	Early to moderate	**Preclinical only**	[141,142,143]
**White Matter Repair**	Clemastine, Anti-LINGO-1	Promote remyelination	Disease-modifying	Early to moderate	**Limited (Phase II)**	[66,125,146]
**Symptomatic**	Cholinesterase inhibitors, Memantine	Neurotransmitter modulation	Symptomatic	Mild to moderate	**Weak (mixed results)**	[135]
**Glymphatic Enhancement**	Sleep optimization, CPAP, Omega-3	**Improve waste clearance**	**Potentially both**	**All stages**	**Emerging**	[158,159,160,161]

CPAP, Continuous positive airway pressure; D + Q, Dasatinib plus Quercetin; GLP-1, Glucagon-like peptide-1; LINGO-1, Leucine-rich repeat and Ig domain-containing Nogo receptor-interacting protein 1; NLRP3, NLR family pyrin domain containing 3; PDE5, Phosphodiesterase type 5; SGLT2, Sodium-glucose cotransporter 2.

## Data Availability

Not applicable.

## Abbreviations

**AD**	Alzheimer’s disease
**VCI**	Vascular cognitive impairment
**VaD**	Vascular Dementia
**SVD**	Small-vessel disease (cerebral)
**BBB**	Blood–brain barrier
**PSD**	Post-stroke dementia
**SIVD**	Subcortical ischemic vascular dementia
**CBF**	Cerebral blood flow
**WMHs**	White-matter hyperintensities
**MRI**	Magnetic-resonance imaging
**CADASIL**	Cerebral autosomal dominant arteriopathy with subcortical infarcts and leukoencephalopathy
**VSMC**	Vascular smooth-muscle cell
**NVU**	Neurovascular unit
**VCAM-1**	Vascular cell adhesion molecule-1
**ICAM-1**	Intercellular adhesion molecule-1
**NO**	Nitric oxide
**eNOS**	endothelial NO synthase
**MMPs**	Matrix metalloproteinases
**ROS**	Reactive oxygen species
**RNS**	Reactive nitrogen species
**NADPH**	Nicotinamide adenine dinucleotide phosphate
**NOX**	NADPH oxidase
**CSF**	Cerebrospinal fluid
**CNS**	Central nervous system
**TNF**	Tumor necrosis factor
**IL**	Interleukin
**NLR**	NOD-like receptor
**NLRP3**	NLR family pyrin domain containing 3
**OPCs**	Oligodendrocyte precursor cells
**AQP4**	Aquaporin-4
**REM**	Rapid eye movement
**DAMPs**	Damage-associated molecular patterns
**DTI**	Diffusion tensor imaging
**APOE**	Apolipoprotein E
**CAA**	Cerebral amyloid angiopathy
**MTHFR**	Methylenetetrahydrofolate reductase
**GWAS**	Genome-wide association study
**FLAIR**	Fluid-attenuated inversion recovery
**SWI**	Susceptibility-weighted imaging
**FA**	Fractional anisotropy
**MD**	Mean diffusivity
**ASL**	Arterial spin labelling
**DSC**	Dynamic susceptibility contrast
**DCE**	Dynamic contrast-enhanced
**NfL**	Neurofilament light chain
**GFAP**	Glial fibrillary acidic protein
**YKL-40**	Chitinase-3-like protein 1
**TREM2**	Triggering Receptor Expressed on Myeloid Cells 2
**DTI-ALPS**	Diffusion Tensor Image Analysis Along the Perivascular Space
**FTD**	Frontotemporal dementia
**SPRINT-MIND**	Systolic Blood Pressure Intervention Trial–Memory and Cognition in Decreased Hypertension
**GLP-1**	Glucagon-like peptide-1
**DASH**	Dietary Approaches to Stop Hypertension
**NMDA**	N-Methyl-D-aspartate
**PDE**	Phosphodiesterase
**AMP**	Adenosine monophosphate
**SASP**	Senescence-associated secretory phenotype
**LINGO-1**	Leucine-rich repeat and Ig domain-containing Nogo receptor-interacting protein 1
**NMN**	Nicotinamide Mononucleotide
**NR**	Nicotinamide Riboside
**CPAP**	Continuous positive airway pressure

## References

[1] World Health Organization (2021). Global Status Report on the Public Health Response to Dementia.

[2] GBD 2019 Dementia Forecasting Collaborators (2022). Estimation of the global prevalence of dementia in 2019 and forecasted prevalence in 2050: An analysis for the Global Burden of Disease Study 2019. Lancet Public Health.

[3] Iadecola C., Duering M., Hachinski V., Joutel A., Pendlebury S.T., Schneider J.A., Dichgans M. (2019). Vascular Cognitive Impairment and Dementia: JACC Scientific Expert Panel. J. Am. Coll. Cardiol..

[4] Wolters F.J., Ikram M.A. (2019). Epidemiology of Vascular Dementia. Arter. Thromb. Vasc. Biol..

[5] Skrobot O.A., O’Brien J., Black S., Chen C., DeCarli C., Erkinjuntti T., Ford G.A., Kalaria R.N., Pantoni L., Pasquier F. (2017). The Vascular Impairment of Cognition Classification Consensus Study. Alzheimer’s Dement..

[6] Román G.C., Tatemichi T.K., Erkinjuntti T., Cummings J.L., Masdeu J.C., Garcia J.H., Amaducci L., Orgogozo J.M., Brun A., Hofman A. (1993). Vascular dementia: Diagnostic criteria for research studies. Report of the NINDS-AIREN International Workshop. Neurology.

[7] Wardlaw J.M., Smith E.E., Biessels G.J., Cordonnier C., Fazekas F., Frayne R., Lindley R.I., O’Brien J.T., Barkhof F., Benavente O.R. (2013). Neuroimaging standards for research into small vessel disease and its contribution to ageing and neurodegeneration. Lancet Neurol..

[8] Kapasi A., DeCarli C., Schneider J.A. (2017). Impact of multiple pathologies on the threshold for clinically overt dementia. Acta Neuropathol..

[9] Boyle P.A., Yu L., Wilson R.S., Leurgans S.E., Schneider J.A., Bennett D.A. (2018). Person-specific contribution of neuropathologies to cognitive loss in old age. Ann. Neurol..

[10] Zlokovic B.V., Gottesman R.F., Bernstein K.E., Seshadri S., McKee A., Snyder H., Greenberg S.M., Yaffe K., Schaffer C.B., Iadecola C. (2020). Vascular contributions to cognitive impairment and dementia (VCID): A report from the 2018 National Heart, Lung, and Blood Institute and National Institute of Neurological Disorders and Stroke Workshop. Alzheimer’s Dement..

[11] Corriveau R.A., Koroshetz W.J., Gladman J.T., Jeon S., Babcock D., Bennett D.A., Carmichael S.T., Dickinson S.L., Dickson D.W., Emr M. (2017). Alzheimer’s Disease-Related Dementias Summit 2016: National Research Priorities. Neurology.

[12] Sweeney M.D., Kisler K., Montagne A., Toga A.W., Zlokovic B.V. (2018). The role of brain vasculature in neurodegenerative disorders. Nat. Neurosci..

[13] Iadecola C. (2013). The pathobiology of vascular dementia. Neuron.

[14] Prins N.D., Scheltens P. (2015). White matter hyperintensities, cognitive impairment and dementia: An update. Nat. Rev. Neurol..

[15] Georgakis M.K., Duering M., Wardlaw J.M., Dichgans M. (2019). WMH and long-term outcomes in ischemic stroke: A systematic review and meta-analysis. Neurology.

[16] Gorelick P.B., Scuteri A., Black S.E., DeCarli C., Greenberg S.M., Iadecola C., Launer L.J., Laurent S., Lopez O.L., Nyenhuis D. (2011). Vascular contributions to cognitive impairment and dementia: A statement for healthcare professionals from the American Heart Association/American Stroke Association. Stroke.

[17] Dichgans M., Leys D. (2017). Vascular Cognitive Impairment. Circ. Res..

[18] Mijajlović M.D., Pavlović A., Brainin M., Heiss W.D., Quinn T.J., Ihle-Hansen H.B., Hermann D.M., Ben Assayag E., Richard E., Thiel A. (2017). Post-stroke dementia—A comprehensive review. BMC Med..

[19] Kalaria R.N. (2016). Neuropathological diagnosis of vascular cognitive impairment and vascular dementia with implications for Alzheimer’s disease. Acta Neuropathol..

[20] Pendlebury S.T., Rothwell P.M. (2009). Prevalence, incidence, and factors associated with pre-stroke and post-stroke dementia: A systematic review and meta-analysis. Lancet Neurol..

[21] Hachinski V. (2015). Stroke and Potentially Preventable Dementias Proclamation: Updated World Stroke Day Proclamation. Stroke.

[22] Levine D.A., Galecki A.T., Langa K.M., Unverzagt F.W., Kabeto M.U., Giordani B., Wadley V.G. (2015). Trajectory of Cognitive Decline After Incident Stroke. JAMA.

[23] Jayaraj R.L., Azimullah S., Beiram R., Jalal F.Y., Rosenberg G.A. (2019). Neuroinflammation: Friend and foe for ischemic stroke. J. Neuroinflammation.

[24] Caplan L.R. (1995). Binswanger’s disease—Revisited. Neurology.

[25] Wardlaw J.M., Smith C., Dichgans M. (2013). Mechanisms of sporadic cerebral small vessel disease: Insights from neuroimaging. Lancet Neurol..

[26] Faraco G., Iadecola C. (2013). Hypertension: A harbinger of stroke and dementia. Hypertension.

[27] Habes M., Erus G., Toledo J.B., Zhang T., Bryan N., Launer L.J., Rosseel Y., Janowitz D., Doshi J., Van der Auwera S. (2016). White matter hyperintensities and imaging patterns of brain ageing in the general population. Brain.

[28] Charidimou A., Boulouis G., Frosch M.P., Baron J.C., Pasi M., Albucher J.F., Banerjee G., Barbato C., Bonneville F., Brandner S. (2022). The Boston criteria version 2.0 for cerebral amyloid angiopathy: A multicentre, retrospective, MRI-neuropathology diagnostic accuracy study. Lancet Neurol..

[29] Allan L.M., Rowan E.N., Firbank M.J., Thomas A.J., Parry S.W., Polvikoski T.M., O’Brien J.T., Kalaria R.N. (2011). Long term incidence of dementia, predictors of mortality and pathological diagnosis in older stroke survivors. Brain.

[30] Brown W.R., Thore C.R. (2011). Review: Cerebral microvascular pathology in ageing and neurodegeneration. Neuropathol. Appl. Neurobiol..

[31] Rutten J.W., Haan J., Terwindt G.M., van Duinen S.G., Boon E.M.J., Lesnik Oberstein S.A.J., Adam M.P., Feldman J., Mirzaa G.M., Pagon R.A., Wallace S.E., Amemiya A. (2000). CADASIL. GeneReviews^®^.

[32] Joutel A., Corpechot C., Ducros A., Vahedi K., Chabriat H., Mouton P., Alamowitch S., Domenga V., Cécillion M., Marechal E. (1996). Notch3 mutations in CADASIL, a hereditary adult-onset condition causing stroke and dementia. Nature.

[33] Wang M.M. (2002). NOTCH3 signaling in vascular smooth muscle cells induces c-FLIP expression via ERK/MAPK activation. J. Biol. Chem..

[34] Rutten J.W., Dauwerse H.G., Gravesteijn G., van Belzen M.J., van der Grond J., Polke J.M., Bernal-Quiros M., Lesnik Oberstein S.A.J. (2016). Archetypal NOTCH3 mutations frequent in public exome: Implications for CADASIL. Ann. Clin. Transl. Neurol..

[35] Ling Y., De Guio F., Duering M., Jouvent E., Hervé D., Godin O., Dichgans M., Chabriat H. (2017). Predictors and Clinical Impact of Incident Lacunes in Cerebral Autosomal Dominant Arteriopathy With Subcortical Infarcts and Leukoencephalopathy. Stroke.

[36] Hara K., Shiga A., Fukutake T., Nozaki H., Miyashita A., Yokoseki A., Kawata H., Koyama A., Arima K., Takahashi T. (2009). Association of HTRA1 mutations and familial ischemic cerebral small-vessel disease. N. Engl. J. Med..

[37] Schaeffer S., Iadecola C. (2021). Revisiting the neurovascular unit. Nat. Neurosci..

[38] Quick S., Moss J., Rajani R.M., Williams A. (2021). A Vessel for Change: Endothelial Dysfunction in Cerebral Small Vessel Disease. Trends Neurosci..

[39] Pober J.S., Sessa W.C. (2014). Inflammation and the blood microvascular system. Cold Spring Harb. Perspect. Biol..

[40] Carare R.O., Bernardes-Silva M., Newman T.A., Page A.M., Nicoll J.A., Perry V.H., Weller R.O. (2008). Solutes, but not cells, drain from the brain parenchyma along basement membranes of capillaries and arteries. Neuropathol. Appl. Neurobiol..

[41] Dhaun N., Webb D.J. (2019). Endothelins in cardiovascular biology and therapeutics. Nat. Rev. Cardiol..

[42] Rosenberg G.A. (2009). Matrix metalloproteinases and their multiple roles in neurodegenerative diseases. Lancet Neurol..

[43] Yang Y., Estrada E.Y., Thompson J.F., Liu W., Rosenberg G.A. (2007). Matrix metalloproteinase-mediated disruption of tight junction proteins in cerebral vessels is reversed by synthetic matrix metalloproteinase inhibitor in focal ischemia in rat. J. Cereb. Blood Flow Metab..

[44] Giannoni P., Arango-Lievano M., Neves I.D., Rousset M.C., Baranger K., Rivera S., Jeanneteau F., Claeysen S., Marchi N. (2016). Cerebrovascular pathology during the progression of experimental Alzheimer’s disease. Neurobiol. Dis..

[45] Ryu J.K., McLarnon J.G. (2009). A leaky blood-brain barrier, fibrinogen infiltration and microglial reactivity in inflamed Alzheimer’s disease brain. J. Cell. Mol. Med..

[46] Wardlaw J.M., Benveniste H., Nedergaard M., Zlokovic B.V., Mestre H., Lee H., Doubal F.N., Brown R., Ramirez J., MacIntosh B.J. (2020). Perivascular spaces in the brain: Anatomy, physiology and pathology. Nat. Rev. Neurol..

[47] Poprac P., Jomova K., Simunkova M., Kollar V., Rhodes C.J., Valko M. (2017). Targeting Free Radicals in Oxidative Stress-Related Human Diseases. Trends Pharmacol. Sci..

[48] Tönnies E., Trushina E. (2017). Oxidative Stress, Synaptic Dysfunction, and Alzheimer’s Disease. J. Alzheimer’s Dis..

[49] Ma M.W., Wang J., Zhang Q., Wang R., Dhandapani K.M., Vadlamudi R.K., Brann D.W. (2017). NADPH oxidase in brain injury and neurodegenerative disorders. Mol. Neurodegener..

[50] Angelova P.R., Abramov A.Y. (2018). Role of mitochondrial ROS in the brain: From physiology to neurodegeneration. FEBS Lett..

[51] Niki E. (2009). Lipid peroxidation: Physiological levels and dual biological effects. Free Radic. Biol. Med..

[52] Singh A., Kukreti R., Saso L., Kukreti S. (2019). Oxidative Stress: A Key Modulator in Neurodegenerative Diseases. Molecules.

[53] Ionescu-Tucker A., Cotman C.W. (2021). Emerging roles of oxidative stress in brain aging and Alzheimer’s disease. Neurobiol. Aging.

[54] Low A., Mak E., Rowe J.B., Markus H.S., O’Brien J.T. (2019). Inflammation and cerebral small vessel disease: A systematic review. Ageing Res. Rev..

[55] ElAli A., Thériault P., Rivest S. (2014). The role of pericytes in neurovascular unit remodeling in brain disorders. Int. J. Mol. Sci..

[56] Prinz M., Jung S., Priller J. (2019). Microglia Biology: One Century of Evolving Concepts. Cell.

[57] Kwon H.S., Koh S.H. (2020). Neuroinflammation in neurodegenerative disorders: The roles of microglia and astrocytes. Transl. Neurodegener..

[58] Escartin C., Galea E., Lakatos A., O’Callaghan J.P., Petzold G.C., Serrano-Pozo A., Blanquer-Rossello M.M., Bribian A., Barcia C., Barbeito L. (2021). Reactive astrocyte nomenclature, definitions, and future directions. Nat. Neurosci..

[59] Kettenmann H., Hanisch U.K., Noda M., Verkhratsky A. (2011). Physiology of microglia. Physiol. Rev..

[60] Verkhratsky A., Nedergaard M. (2018). Physiology of Astroglia. Physiol. Rev..

[61] Voet S., Srinivasan S., Lamkanfi M., van Loo G. (2019). Inflammasomes in neuroinflammatory and neurodegenerative diseases. EMBO Mol. Med..

[62] Greenberg S.M., Bacskai B.J., Hernandez-Guillamon M., Pruzin J., Sperling R., van Veluw S.J. (2020). Cerebral amyloid angiopathy and Alzheimer disease—One peptide, two pathways. Nat. Rev. Neurol..

[63] Rivera E.J., Goldin A., Fulmer N., Tavares R., Wands J.R., de la Monte S.M. (2005). Insulin and insulin-like growth factor expression and function deteriorate with progression of Alzheimer’s disease. J. Alzheimer’s Dis..

[64] Matute C., Torre I., Pérez-Cerdá F., Pérez-Samartín A., Alberdi E., Etxebarria E., Arranz A.M., Ravid R., Rodríguez-Antigüedad A., Sánchez-Gómez M. (2007). P2X(7) receptor blockade prevents ATP excitotoxicity in oligodendrocytes and ameliorates experimental autoimmune encephalomyelitis. J. Neurosci..

[65] Franklin R.J.M., Ffrench-Constant C. (2017). Regenerating CNS myelin—From mechanisms to experimental medicines. Nat. Rev. Neurosci..

[66] Kuhn S., Gritti L., Crooks D., Dombrowski Y. (2019). Oligodendrocytes in Development, Myelin Generation and Beyond. Cells.

[67] Pasi M., Cordonnier C. (2020). Clinical Relevance of Cerebral Small Vessel Diseases. Stroke.

[68] Nedergaard M. (2013). Garbage truck of the brain. Science.

[69] Iliff J.J., Wang M., Liao Y., Plogg B.A., Peng W., Gundersen G.A., Benveniste H., Vates G.E., Deane R., Goldman S.A. (2012). A paravascular pathway facilitates CSF flow through the brain parenchyma and the clearance of interstitial solutes, including amyloid β. Sci. Transl. Med..

[70] Mestre H., Hablitz L.M., Xavier A.L., Feng W., Zou W., Pu T., Monai H., Murlidharan G., Castellanos Rivera R.M., Simon M.J. (2018). Aquaporin-4-dependent glymphatic solute transport in the rodent brain. eLife.

[71] Kress B.T., Iliff J.J., Xia M., Wang M., Wei H.S., Zeppenfeld D., Xie L., Kang H., Xu Q., Liew J.A. (2014). Impairment of paravascular clearance pathways in the aging brain. Ann. Neurol..

[72] Rasmussen M.K., Mestre H., Nedergaard M. (2018). The glymphatic pathway in neurological disorders. Lancet Neurol..

[73] Xie L., Kang H., Xu Q., Chen M.J., Liao Y., Thiyagarajan M., O’Donnell J., Christensen D.J., Nicholson C., Iliff J.J. (2013). Sleep drives metabolite clearance from the adult brain. Science.

[74] Fultz N.E., Bonmassar G., Setsompop K., Stickgold R.A., Rosen B.R., Polimeni J.R., Lewis L.D. (2019). Coupled electrophysiological, hemodynamic, and cerebrospinal fluid oscillations in human sleep. Science.

[75] Tarasoff-Conway J.M., Carare R.O., Osorio R.S., Glodzik L., Butler T., Fieremans E., Axel L., Rusinek H., Nicholson C., Zlokovic B.V. (2015). Clearance systems in the brain-implications for Alzheimer disease. Nat. Rev. Neurol..

[76] Benveniste H., Liu X., Koundal S., Sanggaard S., Lee H., Wardlaw J. (2019). The glymphatic system and waste clearance with brain aging: A review. Gerontology.

[77] Taoka T., Masutani Y., Kawai H., Nakane T., Matsuoka K., Yasuno F., Kishimoto T., Naganawa S. (2017). Evaluation of glymphatic system activity with the diffusion MR technique: Diffusion tensor image analysis along the perivascular space (DTI-ALPS) in Alzheimer’s disease cases. Jpn. J. Radiol..

[78] Wardlaw J.M., Smith C., Dichgans M. (2019). Small vessel disease: Mechanisms and clinical implications. Lancet Neurol..

[79] Nation D.A., Sweeney M.D., Montagne A., Sagare A.P., D’Orazio L.M., Pachicano M., Sepehrband F., Nelson A.R., Buennagel D.P., Harrington M.G. (2019). Blood-brain barrier breakdown is an early biomarker of human cognitive dysfunction. Nat. Med..

[80] Sweeney M.D., Sagare A.P., Zlokovic B.V. (2018). Blood-brain barrier breakdown in Alzheimer disease and other neurodegenerative disorders. Nat. Rev. Neurol..

[81] Montagne A., Barnes S.R., Sweeney M.D., Halliday M.R., Sagare A.P., Zhao Z., Toga A.W., Jacobs R.E., Liu C.Y., Amezcua L. (2015). Blood-brain barrier breakdown in the aging human hippocampus. Neuron.

[82] Zhang C.E., Wong S.M., van de Haar H.J., Staals J., Jansen J.F., Jeukens C.R., Hofman P.A., van Oostenbrugge R.J., Backes W.H. (2017). Blood-brain barrier leakage is more widespread in patients with cerebral small vessel disease. Neurology.

[83] Heneka M.T., Carson M.J., El Khoury J., Landreth G.E., Brosseron F., Feinstein D.L., Jacobs A.H., Wyss-Coray T., Vitorica J., Ransohoff R.M. (2015). Neuroinflammation in Alzheimer’s disease. Lancet Neurol..

[84] Lawrence A.J., Chung A.W., Morris R.G., Markus H.S., Barrick T.R. (2014). Structural network efficiency is associated with cognitive impairment in small-vessel disease. Neurology.

[85] Heneka M.T., McManus R.M., Latz E. (2018). Inflammasome signalling in brain function and neurodegenerative disease. Nat. Rev. Neurosci..

[86] Khalil M., Teunissen C.E., Otto M., Piehl F., Sormani M.P., Gattringer T., Barro C., Kappos L., Comabella M., Fazekas F. (2018). Neurofilaments as biomarkers in neurological disorders. Nat. Rev. Neurol..

[87] Fornari E., Maeder P., Meuli R., Ghika J., Knyazeva M.G. (2012). Demyelination of superficial white matter in early Alzheimer’s disease: A magnetization transfer imaging study. Neurobiol. Aging.

[88] Fernando M.S., Simpson J.E., Matthews F., Brayne C., Lewis C.E., Barber R., Kalaria R.N., Forster G., Esteves F., Wharton S.B. (2006). White matter lesions in an unselected cohort of the elderly: Molecular pathology suggests origin from chronic hypoperfusion injury. Stroke.

[89] Baker D.J., Wijshake T., Tchkonia T., LeBrasseur N.K., Childs B.G., van de Sluis B., Kirkland J.L., van Deursen J.M. (2011). Clearance of p16Ink4a-positive senescent cells delays ageing-associated disorders. Nature.

[90] Montagne A., Zhao Z., Zlokovic B.V. (2017). Alzheimer’s disease: A matter of blood-brain barrier dysfunction?. J. Exp. Med..

[91] Lambert J.C., Ibrahim-Verbaas C.A., Harold D., Naj A.C., Sims R., Bellenguez C., DeStafano A.L., Bis J.C., Beecham G.W., Grenier-Boley B. (2013). Meta-analysis of 74,046 individuals identifies 11 new susceptibility loci for Alzheimer’s disease. Nat. Genet..

[92] Schilling S., DeStefano A.L., Sachdev P.S., Choi S.H., Mather K.A., DeCarli C.D., Wen W., Høgh P., Raz N., Au R. (2013). APOE genotype and MRI markers of cerebrovascular disease: Systematic review and meta-analysis. Neurology.

[93] Montagne A., Nation D.A., Sagare A.P., Barisano G., Sweeney M.D., Chakhoyan A., Pachicano M., Joe E., Nelson A.R., D’Orazio L.M. (2020). APOE4 leads to blood-brain barrier dysfunction predicting cognitive decline. Nature.

[94] Achariyar T.M., Li B., Peng W., Verghese P.B., Shi Y., McConnell E., Benraiss A., Kasper T., Song W., Takano T. (2016). Glymphatic distribution of CSF-derived apoE is isoform specific and suppressed during sleep deprivation. Mol. Neurodegener..

[95] Liu H., Yang M., Li G.M., Qiu Y., Zheng J., Du X., Wang J.L., Liu R.W. (2010). The MTHFR C677T polymorphism contributes to an increased risk for vascular dementia: A meta-analysis. J. Neurol. Sci..

[96] Swardfager W., Lanctôt K., Rothenburg L., Wong A., Cappell J., Herrmann N. (2010). A meta-analysis of cytokines in Alzheimer’s disease. Biol. Psychiatry.

[97] Traylor M., Malik R., Nalls M.A., Cotlarciuc I., Radmanesh F., Thorleifsson G., Hanscombe K.B., Langefeld C., Saleheen D., Rost N.S. (2017). Genetic variation at 16q24.2 is associated with small vessel stroke. Ann. Neurol..

[98] Georgakis M.K., Gill D., Rannikmäe K., Traylor M., Anderson C.D., Lee J.M., Kamatani Y., Hopewell J.C., Worrall B.B., Bernhagen J. (2019). Genetically Determined Levels of Circulating Cytokines and Risk of Stroke. Circulation.

[99] Wardlaw J.M., Doubal F.N., Valdes-Hernandez M., Wang X., Chappell F.M., Shuler K., Armitage P.A., Carpenter T.C., Dennis M.S. (2013). Blood-brain barrier permeability and long-term clinical and imaging outcomes in cerebral small vessel disease. Stroke.

[100] Duering M., Finsterwalder S., Baykara E., Tuladhar A.M., Gesierich B., Konieczny M.J., Malik R., Franzmeier N., Ewers M., Jouvent E. (2018). Free water determines diffusion alterations and clinical status in cerebral small vessel disease. Alzheimer’s Dement..

[101] Maniega S.M., Valdés Hernández M.C., Clayden J.D., Royle N.A., Murray C., Morris Z., Aribisala B.S., Gow A.J., Starr J.M., Bastin M.E. (2015). White matter hyperintensities and normal-appearing white matter integrity in the aging brain. Neurobiol. Aging.

[102] de Guio F., Jouvent E., Biessels G.J., Black S.E., Brayne C., Chen C., Cordonnier C., De Leeuw F.E., Dichgans M., Doubal F. (2016). Reproducibility and variability of quantitative magnetic resonance imaging markers in cerebral small vessel disease. J. Cereb. Blood Flow. Metab..

[103] Zetterberg H. (2016). Neurofilament Light: A Dynamic Cross-Disease Fluid Biomarker for Neurodegeneration. Neuron.

[104] Simrén J., Ashton N.J., Blennow K., Zetterberg H. (2020). An update on fluid biomarkers for neurodegenerative diseases: Recent success and challenges ahead. Curr. Opin. Neurobiol..

[105] Miners J.S., Kehoe P.G., Love S., Zetterberg H., Blennow K. (2019). CSF evidence of pericyte damage in Alzheimer’s disease is associated with markers of blood-brain barrier dysfunction and disease pathology. Alzheimer’s Res. Ther..

[106] Charidimou A., Boulouis G., Gurol M.E., Ayata C., Bacskai B.J., Frosch M.P., Viswanathan A., Greenberg S.M. (2017). Emerging concepts in sporadic cerebral amyloid angiopathy. Brain.

[107] Thrippleton M.J., Backes W.H., Sourbron S., Ingrisch M., van Osch M.J.P., Dichgans M., Fazekas F., Ropele S., Frayne R., van Oostenbrugge R.J. (2019). Quantifying blood-brain barrier leakage in small vessel disease: Review and consensus recommendations. Alzheimer’s Dement..

[108] Yokota H., Vijayasarathi A., Cekic M., Hirata Y., Linetsky M., Ho M., Kim W., Salamon N. (2019). Diagnostic performance of glymphatic system evaluation using diffusion tensor imaging in idiopathic normal pressure hydrocephalus and mimickers. Curr. Gerontol. Geriatr. Res..

[109] van Leijsen E.M.C., de Leeuw F.E., Tuladhar A.M. (2017). Disease progression and regression in sporadic small vessel disease-insights from neuroimaging. Clin. Sci..

[110] Shi Y., Thrippleton M.J., Makin S.D., Marshall I., Geerlings M.I., de Craen A.J.M., van Buchem M.A., Wardlaw J.M. (2016). Cerebral blood flow in small vessel disease: A systematic review and meta-analysis. J. Cereb. Blood Flow Metab..

[111] Benjamin P., Trippier S., Lawrence A.J., Lambert C., Zeestraten E., Williams O.A., Patel B., Morris R.G., Barrick T.R., MacKinnon A.D. (2018). Lacunar infarcts, but not perivascular spaces, are predictors of cognitive decline in cerebral small-vessel disease. Stroke.

[112] Duering M., Righart R., Csanadi E., Jouvent E., Hervé D., Chabriat H., Dichgans M. (2012). Incident subcortical infarcts induce focal thinning in connected cortical regions. Neurology.

[113] Debette S., Schilling S., Duperron M.G., Larsson S.C., Markus H.S. (2019). Clinical significance of magnetic resonance imaging markers of vascular brain injury: A systematic review and meta-analysis. JAMA Neurol..

[114] Janelidze S., Mattsson N., Palmqvist S., Smith R., Beach T.G., Serrano G.E., Chai X., Proctor N.K., Eichenlaub U., Zetterberg H. (2020). Plasma P-tau181 in Alzheimer’s disease: Relationship to other biomarkers, differential diagnosis, neuropathology and longitudinal progression to Alzheimer’s dementia. Nat. Med..

[115] Croall I.D., Lohner V., Moynihan B., Khan U., Hassan A., O’Brien J.T., Morris R.G., Tozer D.J., Cambridge V.C., Harkness K. (2017). Using DTI to assess white matter microstructure in cerebral small vessel disease (SVD) in multicentre studies. Clin. Sci..

[116] Wardlaw J.M., Valdés Hernández M.C., Muñoz-Maniega S. (2015). What are white matter hyperintensities made of? Relevance to vascular cognitive impairment. J. Am. Heart Assoc..

[117] Ashton N.J., Janelidze S., Al Khleifat A., Leuzy A., van der Ende E.L., Karikari T.K., Benedet A.L., Pascoal T.A., Lleó A., Parnetti L. (2021). A multicentre validation study of the diagnostic value of plasma neurofilament light. Nat. Commun..

[118] Rhodius-Meester H.F.M., Benedictus M.R., Wattjes M.P., Barkhof F., Scheltens P., Muller M., van der Flier W.M. (2017). MRI visual ratings of brain atrophy and white matter hyperintensities across the spectrum of cognitive decline are differently affected by age and diagnosis. Front. Aging Neurosci..

[119] Heye A.K., Thrippleton M.J., Armitage P.A., Valdés Hernández M.D.C., Makin S.D., Glatz A., Sakka E., Wardlaw J.M. (2016). Tracer kinetic modelling for DCE-MRI quantification of subtle blood-brain barrier permeability. Neuroimage.

[120] Mattsson N., Andreasson U., Zetterberg H., Blennow K. (2017). Alzheimer’s Disease Neuroimaging Initiative. Association of plasma neurofilament light with neurodegeneration in patients with Alzheimer disease. JAMA Neurol..

[121] Jellinger K.A. (2013). Pathology and pathogenesis of vascular cognitive impairment-a critical update. Front. Aging Neurosci..

[122] Rosenberg G.A., Wallin A., Wardlaw J.M., Markus H.S., Montaner J., Wolfson L., Iadecola C., Zlokovic B.V., Joutel A., Dichgans M. (2016). Consensus statement for diagnosis of subcortical small vessel disease. J. Cereb. Blood Flow Metab..

[123] Benedet A.L., Milà-Alomà M., Vrillon A., Ashton N.J., Pascoal T.A., Lussier F., Karikari T.K., Hourregue C., Cognat E., Dumurgier J. (2021). Differences between plasma and cerebrospinal fluid glial fibrillary acidic protein levels across the Alzheimer disease continuum. JAMA Neurol..

[124] Ringstad G., Valnes L.M., Dale A.M., Pripp A.H., Vatnehol S.A.S., Emblem K.E., Mardal K.A., Eide P.K. (2018). Brain-wide glymphatic enhancement and clearance in humans assessed with MRI. JCI Insight.

[125] Plemel J.R., Liu W.Q., Yong V.W. (2017). Remyelination therapies: A new direction and challenge in multiple sclerosis. Nat. Rev. Drug Discov..

[126] Hampel H., O’Bryant S.E., Molinuevo J.L., Zetterberg H., Masters C.L., Lista S., Kiddle S.J., Batrla R., Blennow K. (2018). Blood-based biomarkers for Alzheimer disease: Mapping the road to the clinic. Nat. Rev. Neurol..

[127] O’Brien J.T., Thomas A. (2015). Vascular dementia. Lancet.

[128] Ngandu T., Lehtisalo J., Solomon A., Levälahti E., Ahtiluoto S., Antikainen R., Bäckman L., Hänninen T., Jula A., Laatikainen T. (2015). A 2 year multidomain intervention of diet, exercise, cognitive training, and vascular risk monitoring versus control to prevent cognitive decline in at-risk elderly people (FINGER): A randomised controlled trial. Lancet.

[129] Hughes D., Judge C., Murphy R., Loughlin E., Costello M., Whiteley W., Bosch J., O’Donnell M.J., Canavan M. (2020). Association of Blood Pressure Lowering With Incident Dementia or Cognitive Impairment: A Systematic Review and Meta-analysis. JAMA.

[130] Williamson J.D., Pajewski N.M., Auchus A.P., Bryan R.N., Chelune G., Cheung A.K., Cleveland M.L., Coker L.H., Crowe M.G., Cushman W.C. (2019). Effect of Intensive vs Standard Blood Pressure Control on Probable Dementia: A Randomized Clinical Trial. JAMA.

[131] Nørgaard C.H., Friedrich S., Hansen C.T., Gerds T., Ballard C., Møller D.V., Knudsen L.B., Kvist K., Zinman B., Holm E. (2022). Treatment with glucagon-like peptide-1 receptor agonists and incidence of dementia: Data from pooled double-blind randomized controlled trials and nationwide disease and prescription registers. Alzheimer’s Dement. Transl. Res. Clin. Interv..

[132] Richardson K., Schoen M., French B., Umscheid C.A., Mitchell M.D., Arnold S.E., Heidenreich P.A., Rader D.J., deGoma E.M. (2013). Statins and cognitive function: A systematic review. Ann. Intern. Med..

[133] Morris M.C., Tangney C.C., Wang Y., Sacks F.M., Barnes L.L., Bennett D.A., Aggarwal N.T. (2015). MIND diet associated with reduced incidence of Alzheimer’s disease. Alzheimer’s Dement..

[134] Bath P.M., Wardlaw J.M. (2015). Pharmacological treatment and prevention of cerebral small vessel disease: A review of potential interventions. Int. J. Stroke.

[135] O’Brien J.T., Erkinjuntti T., Reisberg B., Roman G., Sawada T., Pantoni L., Bowler J.V., Ballard C., DeCarli C., Gorelick P.B. (2003). Vascular cognitive impairment. Lancet Neurol..

[136] Shinohara Y., Katayama Y., Uchiyama S., Yamaguchi T., Handa S., Matsuoka K., Ohashi Y., Tanahashi N., Yamamoto H., Genka C. (2010). Cilostazol for prevention of secondary stroke (CSPS 2): An aspirin-controlled, double-blind, randomised non-inferiority trial. Lancet Neurol..

[137] Kim B.C., Youn Y.C., Jeong J.H., Han H.J., Kim J.H., Lee J., Lee H.S., Lee J.Y., Park K.W., Kim S. (2022). Cilostazol Versus Aspirin on White Matter Changes in Cerebral Small Vessel Disease: A Randomized Controlled Trial. Stroke.

[138] Sanders D.W., Jumper C.C., Ackerman P.J., Bracha D., Donlic A., Kim H., Kenney D., Castello-Serrano I., Suzuki S., Tamura T. (2020). Phosphodiesterase Inhibitors for Alzheimer’s Disease: A Systematic Review of Clinical Trials and Epidemiology with a Mechanistic Rationale. J. Alzheimer’s Dis. Rep..

[139] Ising C., Venegas C., Zhang S., Scheiblich H., Schmidt S.V., Vieira-Saecker A., Schwartz S., Albasset S., McManus R.M., Tejera D. (2019). NLRP3 inflammasome activation drives tau pathology. Nature.

[140] Fagan S.C., Waller J.L., Nichols F.T., Edwards D.J., Pettigrew L.C., Clark W.M., Hall C.E., Switzer J.A., Ergul A., Hess D.C. (2010). Minocycline to improve neurologic outcome in stroke (MINOS): A dose-finding study. Stroke.

[141] Xu M., Pirtskhalava T., Farr J.N., Weigand B.M., Palmer A.K., Weivoda M.M., Inman C.L., Ogrodnik M.B., Hachfeld C.M., Fraser D.G. (2018). Senolytics improve physical function and increase lifespan in old age. Nat. Med..

[142] Zhang P., Kishimoto Y., Grammatikakis I., Gottimukkala K., Cutler R.G., Zhang S., Abdelmohsen K., Bohr V.A., Misra Sen J., Gorospe M. (2019). Senolytic therapy alleviates Aβ-associated oligodendrocyte progenitor cell senescence and cognitive deficits in an Alzheimer’s disease model. Nat. Neurosci..

[143] Gonzales M.M., Garbarino V.R., Marques Zilli E., Petersen R.C., Kirkland J.L., Tchkonia T., Musi N., Seshadri S., Craft S., Orr M.E. (2022). Senolytic Therapy to Modulate the Progression of Alzheimer’s Disease (SToMP-AD): A Pilot Clinical Trial. J. Prev. Alzheimer’s Dis..

[144] Hayden M.R., Grant D.G., Aroor A.R., DeMarco V.G. (2019). Empagliflozin ameliorates type 2 diabetes-induced ultrastructural remodeling of the neurovascular unit and neuroglia in the female db/db mouse. Brain Sci..

[145] Zinman B., Wanner C., Lachin J.M., Fitchett D., Bluhmki E., Hantel S., Mattheus M., Devins T., Johansen O.E., Woerle H.J. (2015). Empagliflozin, cardiovascular outcomes, and mortality in type 2 diabetes. N. Engl. J. Med..

[146] Green A.J., Gelfand J.M., Cree B.A., Bevan C., Boscardin W.J., Mei F., Inman J., Arnow S., Devereux M., Abounasr A. (2017). Clemastine fumarate as a remyelinating therapy for multiple sclerosis (ReBUILD): A randomised, controlled, double-blind, crossover trial. Lancet.

[147] Athauda D., Foltynie T. (2015). The ongoing pursuit of neuroprotective therapies in Parkinson disease. Nat. Rev. Neurol..

[148] Campbell J.M., Bellman S.M., Stephenson M.D., Lisy K. (2017). Metformin reduces all-cause mortality and diseases of ageing independent of its effect on diabetes control: A systematic review and meta-analysis. Ageing Res. Rev..

[149] Sluggett J.K., Koponen M., Bell J.S., Taipale H., Tanskanen A., Tiihonen J., Uusitupa M., Tolppanen A.M., Hartikainen S. (2020). Metformin and risk of Alzheimer’s disease among community-dwelling people with diabetes: A national case-control study. J. Clin. Endocrinol. Metab..

[150] Verdin E. (2015). NAD+ in aging, metabolism, and neurodegeneration. Science.

[151] Das A., Huang G.X., Bonkowski M.S., Longchamp A., Li C., Schultz M.B., Kim L.J., Osborne B., Joshi S., Lu Y. (2018). Impairment of an endothelial NAD+-H2S signaling network is a reversible cause of vascular aging. Cell.

[152] Martens C.R., Denman B.A., Mazzo M.R., Armstrong M.L., Reisdorph N., McQueen M.B., Chonchol M., Seals D.R. (2018). Chronic nicotinamide riboside supplementation is well-tolerated and elevates NAD+ in healthy middle-aged and older adults. Nat. Commun..

[153] Skrobot O.A., Black S.E., Chen C., DeCarli C., Erkinjuntti T., Ford G.A., Kalaria R.N., O’Brien J., Pantoni L., Pasquier F. (2018). Progress toward standardized diagnosis of vascular cognitive impairment: Guidelines from the Vascular Impairment of Cognition Classification Consensus Study. Alzheimer’s Dement..

[154] Smith E.E., Cieslak A., Barber P., Chen J., Chen Y.W., Donnini I., Edwards J.D., Frayne R., Field T.S., Hegedus J. (2017). Therapeutic strategies and drug development for vascular cognitive impairment. J. Am. Heart Assoc..

[155] Lipsman N., Meng Y., Bethune A.J., Huang Y., Lam B., Masellis M., Herrmann N., Heyn C., Aubert I., Boutet A. (2018). Blood-brain barrier opening in Alzheimer’s disease using MR-guided focused ultrasound. Nat. Commun..

[156] Livingston G., Huntley J., Sommerlad A., Ames D., Ballard C., Banerjee S., Brayne C., Burns A., Cohen-Mansfield J., Cooper C. (2020). Dementia prevention, intervention, and care: 2020 report of the *Lancet* Commission. Lancet.

[157] Kivipelto M., Mangialasche F., Ngandu T. (2018). Lifestyle interventions to prevent cognitive impairment, dementia and Alzheimer disease. Nat. Rev. Neurol..

[158] Hablitz L.M., Plá V., Giannetto M., Vinitsky H.S., Stæger F.F., Metcalfe T., Nguyen R., Benrais A., Nedergaard M. (2020). Circadian control of brain glymphatic and lymphatic fluid flow. Nat. Commun..

[159] Ju Y.E., Finn M.B., Sutphen C.L., Herries E.M., Jerome G.M., Ladenson J.H., Crimmins D.L., Fagan A.M., Holtzman D.M. (2016). Obstructive sleep apnea decreases central nervous system-derived proteins in the cerebrospinal fluid. Ann. Neurol..

[160] Ren H., Luo C., Feng Y., Yao X., Shi Z., Liang F., Kang J.X., Wan J.B., Pei Z., Su H. (2017). Omega-3 polyunsaturated fatty acids promote amyloid-β clearance from the brain through mediating the function of the glymphatic system. FASEB J..

[161] Lundgaard I., Lu M.L., Yang E., Peng W., Mestre H., Hitomi E., Deane R., Nedergaard M. (2017). Glymphatic clearance controls state-dependent changes in brain lactate concentration. J. Cereb. Blood Flow Metab..

[162] Hampel H., Toschi N., Babiloni C., Baldacci F., Black K.L., Bokde A.L.W., Bun R.S., Cacciola F., Cavedo E., Chiesa P.A. (2018). Revolution of Alzheimer precision neurology. Passageway of systems biology and neurophysiology. J. Alzheimer’s. Dis..

[163] Blair G.W., Appleton J.P., Flaherty K., Doubal F., Sprigg N., Dooley R., Richardson C., Hamilton I., Law Z.K., Shi Y. (2019). Tolerability, safety and intermediary pharmacological effects of cilostazol and isosorbide mononitrate, alone and combined, in patients with lacunar ischaemic stroke: The LACunar Intervention-1 (LACI-1) trial, a randomised clinical trial. eClinicalMedicine.

[164] Amor S., Puentes F., Baker D., van der Valk P. (2010). Inflammation in neurodegenerative diseases. Immunology.

[165] Biessels G.J., Despa F. (2018). Cognitive decline and dementia in diabetes mellitus: Mechanisms and clinical implications. Nat. Rev. Endocrinol..

[166] Iadecola C., Gottesman R.F. (2019). Neurovascular and cognitive dysfunction in hypertension. Circ. Res..

[167] Shi Y., Wardlaw J.M. (2016). Update on cerebral small vessel disease: A dynamic whole-brain disease. Stroke Vasc. Neurol..

